# Shaping ability of Reciproc Blue reciprocating instruments with or without glide path in simulated S-shaped root canals

**DOI:** 10.15171/joddd.2018.010

**Published:** 2018-03-14

**Authors:** Cangul Keskin, Evren Sarıyılmaz, Murat Demiral

**Affiliations:** ^1^Ondokuz Mayıs University, Department of Endodontics, Faculty of Dentistry, Samsun, Turkey; ^2^Ordu University, Department of Endodontics, Faculty of Dentistry, Ordu, Turkey; ^3^Çekmekoy Oral Health Center, Istanbul, Turkey

**Keywords:** Root canal preparation, Glide path, Nickel-titanium instruments

## Abstract

***Background.*** The present study aimed to compare the shaping ability of Reciproc Blue instruments with or without the creation of a glide path in simulated S-shaped root canals.

***Methods.*** Root canals of thirty #15.02 clear resin S-shaped blocks were dyed using ink and photographed. Then the blocks were randomly divided into 2 groups: group A: Reciproc Blue with glide path created with ProGlider and group B: Reciproc Blue with no glide path preparation (n=15). The blocks were also photographed after preparation. The pre- and post-prepara-tion images were superimposed and evaluated at 9 different measurement points according to the 3 zones, as coronal straight, first curvature, and apical curvature zones. The data were evaluated with independent t-test or Kruskal-Wallis tests with 5% significance interval.

***Results.*** Group B removed greater amount of material from the inner aspect of simulated canal at the first curvature and apical curvature zones and from the outer aspect of the canal at apical curvature zone (P<0.05). Both groups exhibited trans-portation and the transportation width in group B was significantly greater in the levels of apical curvature zone (P<0.05).

***Conclusion.*** Glide path preparation using ProGlider rotary instrument improved the shaping ability of Reciproc Blue R25 instrument by leading to less transportation and maintaining centering ability.

## Introduction

Glide path preparation provides guidance for root canal preparation instruments to follow.^1,2^ Glide path can be described as a smooth and centered preparation from root canal orifice to the apical foramen.^1,2^ An ideal glide path has been reported to prevent difficulties and complications that might arise during chemomechanical preparation such as taper lock, ledge formation, transportation and instrument fracture.^1,3,4^ The use of stainless steel K-files or motor driven nickel-titanium (NiTi) instruments have been suggested for glide path preparation.^5-8^ The use of motor driven NiTi rotary instruments has been reported to provide a less time-consuming and safe glide path preparation, respecting the original canal anatomy.^5,9^

ProGlider (Dentsply Sirona, Ballaigues, Switzerland) is a rotary glide path instrument and is manufactured from memory NiTi wire (M-wire). M-wire provides increased cyclic fatigue resistance compared to the conventional NiTi alloys.^10^ The ProGlider instrument has a tip diameter of 0.16 at D0, with progressive taper ranging from 2% to 8% and square cross-sectional shape.^9^

In 2008, a new approach to use ProTaper F2 instrument (Dentsply Maillefer, Ballaigues, Switzerland) in reciprocating motion was proposed, leading to a new perspective of NiTi instrument kinematics.^11^ Reciprocation motion was reported to increase the life span of instruments by exposing the instrument to lower stress values than continuous rotation.^11,12^ Reciproc Blue (VDW, Munich, Germany) is a reciprocating instrument, which received an innovative heat treatment to transform the molecular structure of the alloy and give the instrument a blue color.^13^ Recent studies showed that thermally treated alloy increased the flexibility and cyclic fatigue resistance of the Reciproc Blue compared to M-wire.^14,15^ However, to the authors’ knowledge no study has evaluated the shaping ability of Reciproc Blue with or without a glide path preparation. The present study aimed to compare shaping ability of Reciproc Blue instruments with or without glide path preparation in simulated S-shaped root canals.

## Methods

The present study used thirty S-shaped Endo Training blocks with a size of #15.02 (Dentsply Sirona). The blocks were numbered and the simulated root canals were dyed by injection of black ink (Pelikan, Istanbul, Turkey). The blocks were photographed using a standardized setup that would provide superimposition of the post-preparation images. The images were saved as JPEG files and transferred to AutoCAD Software (Autodesk, CA, USA). Then, the simulated root canals were flushed with distilled water to remove the ink and the blocks were randomly divided into 2 groups (n=15).

**Group A:** The simulated S-shaped canals were prepared with ProGlider at 300 rpm and 500 gcm-^1^ torque to full working length using endomotor (VDW Gold, VDW, Munich, Germany). Then Reciproc Blue R25 instrument was used by the same endomotor (VDW, Munich) in “Reciproc All” mode. The instrument was introduced into the canal with a light and slow in-and-out pecking motion, with an amplitude of 3 mm. Following 3 pecking motions, the instrument was pulled out and the flutes were cleaned. The root canals were flushed with distilled water between each instrument use. The instrumentation was carried out until the working length was reached.

**Group B:** The simulated canals were prepared using Reciproc Blue R25 just as described in group A without glide path preparation.

All the preparation steps were performed by an experienced endodontist. Each instrument was used for preparation of a single block. A total of 20 mL of distilled water was used for irrigation in both groups.

**Figure 1:**
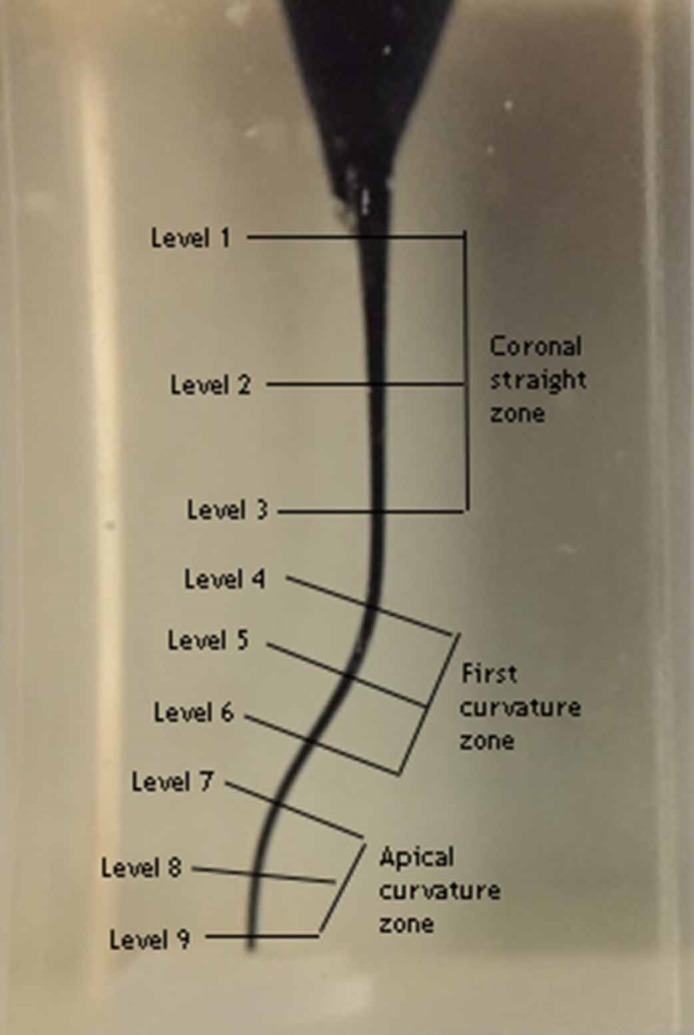
Representative image of the defined levels of 9 measuring points and the 3 zones containing them.

Then the root canals were dyed with red ink (Pelikan, Istanbul, Turkey) using a syringe. The post-preparation images were taken and saved as JPEG files. Pre- and post-preparation images were transferred to an image analysis software (AutoCAD, Autodesk, San Rafael, CA, USA) for superimposition. Nine measurement points were detected along the length of the canals and evaluated by dividing into 3 zones, as levels 1‒3 at coronal straight zone (CS), levels 4‒6 at first curvature zone (FC) and levels 7‒9 at apical curvature zone (AC) (Figure 1). The amount of the resin removed from the inner and outer sides of the canals was measured by AutoCAD. The amount of transportation was calculated as the absolute value of the difference between the amount of resins removed from inner and outer aspect of canals. Centering ratio was calculated using the amount of resin removed from 2 aspects of canal by dividing the smaller to the greater one. Data were analyzed using independent t-test or Kruskal-Wallis tests according to the normality of data distribution with 5% significance threshold using SPSS (IBM, SPSS Inc, Chicago, IL, USA).

## Results

**Figure 2:**
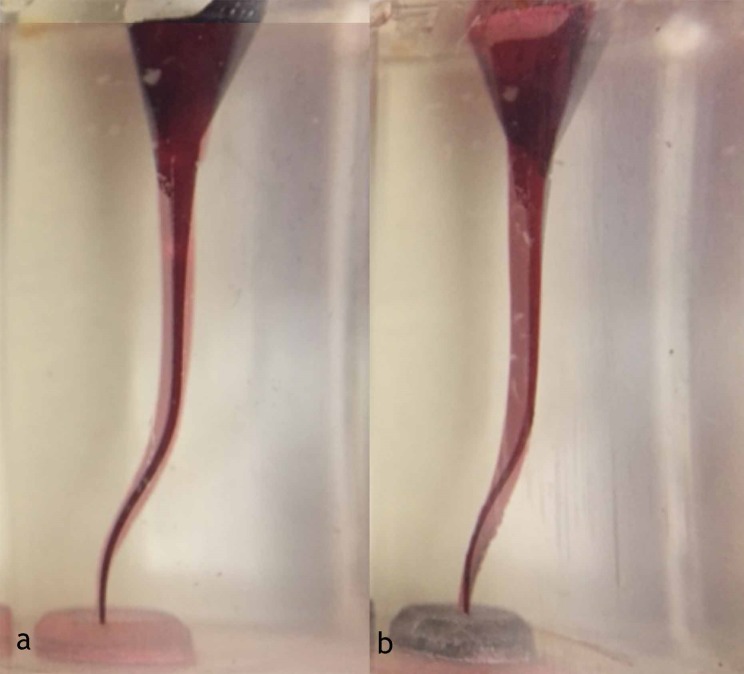
Representative appearance of superimposed pre- and post-preparation images (a) ProGlider and Reciproc Blue group (b) Reciproc Blue group.

Figure 2 illustrates the representative samples of each group. One instrument from group B separated during preparation. No other canal aberrations occurred. Table 1 presents the mean amount of material removed from the inner and outer aspects of the root canals. Independent sample t-test revealed that at all the levels, group B (Reciproc Blue) removed greater amount of material from the inner aspect of simulated root canals than group A (ProGlider + Reciproc Blue). However, the differences were statistically significant only at 3 levels, which were levels 5 and 6 from the FC zone and level 9 from AC zone (P<0.05). Similarly, group B removed greater amount of resin material from the outer aspect of the root canals than group A. At this time, the differences were statistically significant at 7 levels of 9, which are presented in Table 1 (P<0.05).

Both groups caused transportation at all the 9 levels (Table 2). The mean transportation values of group B were greater than group A at all the levels. Kruskal-Wallis test revealed that the differences were statistically significant at 3 levels, as level 1 from the CS zone, and levels 8 and 9 from the AC zone (P<0.05).

Table 3 presents the means and standard deviations of centering ratios of groups A and B. Significant greater values were detected at 4 of 9 levels of group A compared to group B regarding the centering ratio (P<0.05).

**Table 1:** Mean and standard deviation values for the amount of removed material detected at 9 different levels (mm)

	**Inner measurements**			**Outer measurements**		
	**ProGlider** **Reciproc Blue**	**Reciproc Blue**	**P-value**	**ProGlider** **Reciproc Blue**	**Reciproc Blue**	**P-value**
**Level 1 (CS)**	0.26±0.05	0.28±0.04	0.448	0.17±0.02	0.22±0.05	0.001*
**Level 2 (CS)**	0.26±0.07	0.27±0.05	0.657	0.15±0.03	0.23±0.11	0.024*
**Level 3 (CS)**	0.22±0.05	0.26±0.10	0.151	0.19±0.04	0.26±0.13	0.048*
**Level 4 (FC)**	0.23±0.04	0.25±0.07	0.319	0.20±0.03	0.25±0.10	0.085
**Level 5 (FC)**	0.29±0.03	0.35±0.09	0.036*	0.16±0.02	0.20±0.11	0.221
**Level 6 (FC**)	0.23±0.07	0.38±0.10	0.000*	0.21±0.06	0.27±0.08	0.045*
**Level 7 (AC)**	0.12±0.06	0.17±0.08	0.153	0.24±0.08	0.34±0.05	0.001*
**Level 8 (AC)**	0.07±0.02	0.09±0.01	0.635	0.21±0.09	0.39±0.03	0.000*
**Level 9 (AC)**	0.06 ±0.03	0.29±0.04	0.000*	0.01±0.00	0.10±0.03	0.000*

P-values marked with * mean statistically significant difference between ProGlider-Reciproc Blue and Reciproc Blue groups (P<0.05). CS, coronal straight zone; FC, first curvature zone; AC, apical curvature zone

**Table 2:** Mean and standard deviation values for the amount of transportation irrespective of the direction at 9 measurement levels (mm)

	**ProGlider + Reciproc Blue**	**Reciproc Blue**	**P-value**
**Level 1 (CS)**	0.04±0.06	0.11±0.05	0.004*
**Level 2 (CS)**	0.04±0.14	0.10±0.07	0.367
**Level 3 (CS)**	0.00±0.21	0.03±0.06	0.599
**Level 4 (FC)**	0.00±0.15	0.00±0.21	0.579
**Level 5 (FC)**	0.13±0.04	0.14±0.19	0.716
**Level 6 (FC)**	0.02±0.11	0.11±0.18	0.216
**Level 7 (AC)**	0.11±0.12	0.17±0.13	0.267
**Level 8 (AC)**	0.12±0.09	0.29±0.04	0.000*
**Level 9 (AC)**	0.06±0.03	0.19±0.05	0.000*

P-values marked with *mean statistically significant difference between ProGlider-Reciproc Blue and Reciproc Blue groups (P<0.05).

**Table 3:** Centering ratio means and standard deviations detected at 9 different levels

	**ProGlider + Reciproc Blue**	**Reciproc Blue**	**P-value**
**Level 1 (CS)**	0.79±0.15	0.60±0.14	0.001*
**Level 2 (CS)**	0.59±0.15	0.60±0.20	0.826
**Level 3 (CS)**	0.77±0.14	0.58±0.25	0.018*
**Level 4 (FC)**	0.82±0.18	0.67±0.22	0.056
**Level 5 (FC)**	0.57±0.10	0.47±0.22	0.165
**Level 6 (FC)**	0.75±0.23	0.68±0.27	0.444
**Level 7 (AC)**	0.54±0.27	0.54±0.33	0.994
**Level 8 (AC)**	0.48±0.25	0.24±0.04	0.001*
**Level 9 (AC)**	0.76±0.16	0.56±0.20	0.001*

P-values marked with *mean statistically significant difference between ProGlider-Reciproc Blue and Reciproc Blue groups(P<0.05).

## Discussion

Natural teeth exhibit a high variability in the dimension, size and shape of the root canal system. The use of resin blocks provides standardization of dimensions of simulated root canals such as length, diameter, curvature angle and radius of curvature. On the other hand, inability to mimic human teeth due to much lower surface hardness compared to dentin and possibility of softening of resin due to friction during preparation constitute the drawbacks of this technique. It was claimed that the softening resin might attach to the blades of the instrument and cause the instrument to deform or separate.^16^ In the present study, one instrument in group B was separated, whereas no other aberrations occurred. Nevertheless, the performance of instruments has been associated with their ability to respect original canal anatomy.^17^ Therefore the resin blocks were preferred in the present study.

Reciprocating movement was developed mainly to reduce torsional stress around an instrument, thereby decreasing the rate of torsional failure.^11^ Reciproc Blue is subjected to an innovative heat treatment that transforms the molecular structure of the alloy and gives the instrument a blue color.^18^ According to the authors’ literature research, the effect of glide path creation on the shaping ability of Reciproc Blue has not been investigated yet. The present study aimed to evaluate the effect of glide path creation on the shaping ability of Reciproc Blue. ProGlider was used for glide path preparation. The results of the present study indicated that the use of ProGlider instrument for glide path preparation improved the shaping ability properties of Reciproc Blue R25 instrument.

In the present study the measurements were conducted at 9 different points, which were grouped into 3 zones according to their location: the coronal straight, the first curvature and the apical curvature. During root canal preparation it is favorable to maintain the original position and shape of apical foramen in order to accomplish an ideal seal.^19,20^ However, all the preparation instruments change the position of root canals. According to the results of the present study, glide path preparation increased the centering ability of Reciproc Blue R25 instruments, especially at the apical curvature zone. The results of the present study are consistent with the reports of Lim et al and Nazarimoghadam et al.^21,22^ De Carvalho et al^23^ reported that reciprocating instruments tended to deviate from the central axis of root canals when no glide path was created.^23^ Decreased centering ratio values in group B, in which the glide path was not prepared, could be attributed to this tendency of instruments.

Superimposed pre- and post-preparation images of prepared S-shaped resin blocks were used in the present study. A limitation of this technique is inability to evaluate the shaping ability three-dimensionally. Micro-computed tomography provides three-dimensional information about the effects of the instrumentation systems. On the other hand, the use of resin blocks is a reproducible and inexpensive technique, which allows two-dimensional quantitative measurements.

## Conclusions

Within the limitations of the present study, it was concluded that the creation of a glide path using ProGlider rotary instruments improved the shaping ability of Reciproc Blue R25 instrument to respect and follow the original canal curvature in S-shaped resin simulated canals. Due to the differences between resin material and the human dentin, further studies are required to evaluate the effects of instruments using human teeth.

## Acknowledgments

None.

## Funding

No funding reported.

## Competing interests

The authors declare that they have no competing interests with regards to authorship and/or publication of this paper.

## Ethics approval

Not applicable.
